# PS2 mRNA expression adds prognostic information to node status for 6-year survival in breast cancer.

**DOI:** 10.1038/bjc.1998.78

**Published:** 1998

**Authors:** A. M. Thompson, R. A. Elton, R. A. Hawkins, U. Chetty, C. M. Steel

**Affiliations:** Department of Surgery, Ninewells Hospital and Medical School, Dundee, UK.

## Abstract

**Images:**


British Journal of Cancer (1998) 77(3), 492–496
© 1998 Cancer Research Campaign

### PS2 mRNA expression adds prognostic information to node status for 6-year survival in breast cancer

**AM Thompson[1], RA Elton[2], RA Hawkins[3], U Chetty[4] and CM Steel[5]**

[1]Department of Surgery, Ninewells Hospital and Medical School, Dundee DD1 9SY, UK; [2]Medical Statistics Unit and [3]Department of Surgery, Royal Infirmary, Lauriston Place, Edinburgh EH3 9YW, UK; [4]Edinburgh Breast Unit, Western General Hospital, Crewe Road, Edinburgh EH4 2XU, UK; [5]Bute Medical Buildings, University of St Andrews, St Andrews KY16 9TS, UK

**Summary** Expression of pS2, an oestrogen-regulated gene, has been associated with a good short-term prognosis and response to endocrine therapy. The aim of this study was to determine whether expression of mRNA for the pS2 gene in breast cancer could contribute useful information on disease behaviour and survival at medium-term follow-up. Northern blotting was used to detect pS2 messenger ribonucleic acid (mRNA) in the primary tumour tissue from each of 90 patients with breast cancer. Axillary node status was established by sampling or clearance, oestrogen receptor concentration by enzyme immunosorbant assay and follow-up was continued for at least 6 years or until death. At 83 months mean follow-up, 29 of 90 (32%) patients had recurrent disease and, of these, 18 (20%) had died from breast cancer. pS2 mRNA expression, present in 26 of 90 (29%) cancers, was associated with freedom from disease recurrence ($P = 0.026$) and was significantly associated with survival at a minimum of 6 years follow-up ($P < 0.001$). Pathological node status and tumour size were also significantly associated with disease recurrence ($P < 0.001$ and $P = 0.002$ respectively) and inversely with survival ($P < 0.001$ and $P < 0.001$ respectively). After multiple Cox regression analysis, pS2 expression was still a significant predictor of recurrence (but not survival) after adjusting for node status and tumour size; oestrogen receptor was an independent predictor of survival. The combination of node status and pS2 expression discriminated patients with particularly good prognosis (node negative, pS2 positive: no mortality at 6 years) or poor prognosis (node positive, pS2 negative; 41% mortality at 6 years). Evaluation of pS2 expression in breast cancer at diagnosis may provide additional useful prognostic information to conventional staging.

**Keywords:** breast cancer; pS2, prognosis

The detection of pS2 expression in response to an oestrogenic stimulus in breast cancer cells (Masiakowski et al, 1982) – an effect that can be antagonized by tamoxifen – led to speculation that pS2 could be an oestrogen-regulated gene of clinical importance. The pS2 gene (also known as BCEI, pNR-2, Md2) encodes a 600-base mRNA (Masiakowski et al, 1982), which is translated to a cysteine-rich protein with structural similarities to insulin-like growth factors I and II (Rio et al, 1987; Stack et al, 1988). The clinical interest in pS2 is the result of an association with oestrogen receptor expression and with other conventional markers of good prognosis (Rio et al, 1987; Stack et al, 1988; Schwartz et al, 1991; Predine et al, 1992; Thompson et al, 1993; Detre et al, 1994; Foekens et al, 1994; Speiser et al, 1994; Gibert et al, 1996; Tutschek et al, 1996). pS2 expression is associated with good prognosis in female – but not in male – patients at short-term follow-up (Foekens et al, 1990; Schwartz et al, 1991; Predine et al, 1992; Gion et al, 1993; Kardas et al, 1993; Thompson et al, 1993; Foekens et al, 1994; Speiser et al, 1994). Conversely, pS2-negative patients have a significantly shorter relapse-free survival (Gion et al, 1993; Foekens et al, 1994; Speiser et al, 1994; Schmidt et al, 1996) and overall survival (Gion et al, 1993; Speiser et al, 1994). pS2 also predicts a subsequent response to hormone manipulation as first-line therapy and on disease relapse (Henry et al, 1988, 1990, 1991; Schwartz et al 1991; Soubeyran et al, 1996). Thus, the detection of pS2 expression in breast cancers may define a subset of cancers with functional oestrogen receptors and hence patients with a good prognosis who are more likely to respond to endocrine manipulation.

The aim of this study was to determine whether pS2 gene mRNA expression could contribute useful information on disease behaviour and survival after a minimum of 6 years (medium term) follow-up.

*Received 3 February 1997*
*Revised 29 May 1997*
*Accepted 25 June 1997*

*Correspondence to:* AM Thompson

### PATIENTS AND METHODS

Ninety female patients with primary, previously untreated, histologically proven, invasive breast cancer (mean age 56 years, range 30–78 years) underwent surgery for breast cancer. The 2-year follow-up on a subgroup of these women forms part of a previous report (Thompson et al, 1993). Patients with impalpable disease or small tumours (when insufficient material was available for study) were excluded from the study. Fifty-six women were post-menopausal.

The tumour size on the resected specimen was measured as less than 2 cm (pT1) in nine patients, 2–4.9 cm (pT2) in 51 patients or greater than 5 cm (pT3) in 30 patients. Pathological axillary node status (based on 2–25 nodes per patient) was determined by axillary node sampling or clearance, with 46 of 90 patients node positive and 44 node negative at the time of diagnosis. The histology of the tumours was invasive ductal cancer of no special type (80 cancers), lobular breast cancer (seven) or special type (three). The

**Figure 1** pS2 mRNA expression in breast cancers, breast cancer cell lines and reduction mammoplasty tissue. Northern blot autoradiographs probed sequentially with pS2 (0.6-kb mRNA, upper panel) and actin (1.8-kb mRNA, internal control for loading, lower panel). a, Breast cancer, node positive, no disease recurrence; b, breast cancer, node positive, no disease recurrence; c, breast cancer, node negative, no disease recurrence; d, reduction mammoplasty tissue; e, breast cancer cell line MDA MB 231 (oestrogen receptor 0 fmol $mg^{-1}$ protein); f, breast cancer cell line MCF 7 (oestrogen receptor 120 fmol $mg^{-1}$ protein); g, breast cancer cell line T 47 D (oestrogen receptor 40 fmol $mg^{-1}$ protein); h, breast cancer, node positive, disease recurrence; i, breast cancer, node positive, disease recurrence; j, breast cancer, node negative, disease recurrence

**Figure 3** pS2 mRNA expression related to disease recurrence. Breast cancer recurrence against time since diagnosis (in months) for patients with pS2 mRNA expression in the primary cancer (—) and patients with no pS2 mRNA in the cancer (- - -), demonstrating a significant association between pS2 mRNA expression ($P < 0.026$) and freedom from disease recurrence

**Figure 2** pS2 mRNA expression and survival in breast cancer. Patient survival against time since diagnosis (in months) for patients with pS2 mRNA expression in the primary cancer (—) and patients with no pS2 mRNA in the cancer (- - -), demonstrating a significant association between pS2 mRNA expression ($P < 0.001$) and survival

**Figure 4** Axillary node metastasis and prognosis in breast cancer. Patient survival against time since diagnosis (in months) for patients subdivided into axillary node negative (• • •), one to three axillary nodes containing tumour (- - -) or four or more axillary nodes involved with tumour (—)

oestrogen receptor content of the tumours was measured using an enzyme immunosorbant assay (ER-EIA, Abbott Laboratories, North Chicago, IL, USA) and expressed in fmol $mg^{-1}$ protein. Tumour oestrogen receptor concentrations of 20 fmol $mg^{-1}$ protein or more was considered oestrogen receptor moderate or rich (Anderson et al, 1989). Tumour grade and progesterone receptor status was not assessed, in keeping with our clinical and laboratory practice at that time.

In addition to surgical treatment, adjuvant therapy was administered to 79 of the 90 women: 66 received 20 mg of tamoxifen for a minimum of 5 years, 11 CMF and two underwent surgical oophorectomy. Clinical and radiological follow-up (including annual mammography) were continued for at least 6 years or until death; follow-up ranged from 5 to 98 months with a mean follow-up of 83 months. Total RNA was extracted from snap-frozen tumour tissue using the lithium chloride–urea method, and pS2 expression was determined using the Northern blot technique, with a cDNA probe (Masaikowski et al, 1982) used to detect pS2 messenger ribonucleic acid (mRNA) and actin (Minty et al, 1981) as an internal control for RNA loading (Thompson et al, 1993).

© Cancer Research Campaign 1998 

**Figure 5** Axillary node metastases and disease-free interval. Disease recurrence plotted against time since diagnosis (in months) for patients subdivided into axillary node negative (• • •), one to three axillary nodes containing tumour (- - -) or four or more axillary nodes involved with tumour (—)

Autoradiographs of the probed Northern blots were examined for tumour tissue from the 90 patients with primary breast cancer, ten patients who had undergone reduction mammoplasty and three breast cancer cell lines: MCF-7, T47D and MDA MB 231.

The chi-square test was used to compare pS2 mRNA expression and oestrogen receptor protein in the tumours and to compare recurrence and death rates at 6 years. Cox proportional hazards regression and multiple Cox regression were used to test whether pS2 mRNA expression, node status, tumour size on pathology measurement, log oestrogen receptor concentration, oestrogen receptor status (low vs moderate/rich) or menopausal status were associated with recurrence of disease or with survival.

### RESULTS

pS2 mRNA expression was detected in 26 of 90 (29%) breast cancers, six of ten reduction mammoplasty specimens and MCF7 and T47D but not the MDA MB 231 cell line (Figure 1). Fifty-one tumours were oestrogen receptor moderate or rich and 39 were oestrogen receptor poor. pS2 mRNA expression was associated with oestrogen receptor concentrations of 20 or greater fmol $mg^{-1}$ protein (chi-square 5.8, $P = 0.015$, 1 d.f., 95% confidence limits for oestrogen receptor 1.24–12.3). There was no association between pS2 expression and node status, tumour size or menopausal status. At a mean follow-up of 84 months, 29 of 90 (32%) patients had developed recurrent disease and 18 of 90 (20%) of these had died from breast cancer. All 26 patients with pS2 mRNA expression in the tumour at diagnosis were alive. On univariate analysis, pS2 mRNA expression was significantly associated with survival ($P < 0.001$) (Figure 2) and with disease-free interval ($P = 0.026$) (Figure 3). Taking the clinically useful cut-off of 20 fmol $mg^{-1}$ protein to discriminate between oestrogen receptor-poor (< 20) and oestrogen receptor-moderate/rich (≥ 20) tumours, or using oestrogen receptor as a continuous variable, or as the log of oestrogen receptor, oestrogen receptor expression was not associated with survival or disease-free interval by univariate analysis.

Axillary node metastasis, detected in 6 of 90 (51%) patients at diagnosis, was significantly associated both with poor prognosis ($P < 0.001$) (Figure 4) and with shorter disease-free interval ($P < 0.001$) (Figure 5). Tumour size, measured by the pathologist on the resected specimen, was also significantly associated with recurrence ($P < 0.001$) and survival ($P = 0.003$).

**Table 1** Multiple Cox regression analysis of clinical and pathological parameters related to disease recurrence and survival in breast cancer

| Factor | *P*-value | | | |
|---|---|---|---|---|
| | Recurrence | | Survival | |
| | Univariate | Multivariate | Univariate | Multivariate |
| Node metastasis | < 0.001 | < 0.001 | 0.001 | < 0.001 |
| Tumour size | < 0.001 | 0.002 | 0.003 | < 0.001 |
| pS2 | 0.026 | 0.020 | < 0.001 | 0.92 |
| Oestrogen receptor | 0.90 | 0.52 | 0.10 | 0.001 |
| Menopausal status | 0.95 | 0.11 | 0.82 | 0.08 |

**Table 2** Recurrence and survival by node status and pS2 mRNA expression.

| | Node negative | | Node positive | | |
|---|---|---|---|---|---|
| | pS2 mRNA+ | pS2 mRNA– | pS2 mRNA+ | pS2 mRNA– | Total |
| Alive and well | 13 | 27 | 10 | 11 | 61 |
| Recurrent disease | 1 | 0 | 2 | 8 | 11 |
| Dead | 0 | 5 | 0 | 13 | 18 |
| Total | 14 | 32 | 12 | 32 | |

pS2 mRNA+, pS2 mRNA positive; pS2 mRNA–, pS2mRNA negative.

© Cancer Research Campaign 1998

By multiple Cox regression (Table 1), node status and tumour size both remained independently significant for both recurrence and survival; pS2 expression was still a significant predictor of recurrence after adjusting for both these variables, but was not a predictor for mortality. Oestrogen receptor status became independently predictive of mortality (but not disease recurrence) after adjusting for nodes and size. The combination of node status and pS2 expression (Table 2) defined patients with particularly good prognosis (node negative, pS2 positive; no mortality at 5 years) or poor prognosis (node positive, pS2 negative; 41% mortality at 5 years). When the cancer did not express pS2 mRNA, patients who were node positive were significantly more likely ($P < 0.001$) to develop disease recurrence than patients who were node negative.

### DISCUSSION

This study examined pS2 mRNA expression in 90 breast cancers and related pS2 expression to clinical and pathological parameters and to disease behaviour at a minimum follow-up of 6 years.

pS2 expression was detected in 29% of the breast cancers studied, which is within the range (22–58%) for mRNA detection in breast cancer reported by others (Rio et al, 1987; Stack et al, 1988; Skilton et al, 1989; Henry et al, 1990; Zaretsky et al, 1990; Hahnel et al, 1991; Delvenne et al, 1992; Wysocki et al, 1994) and similar to the range of 27–68% of breast cancers that express pS2 protein (Foekens et al, 1990; Henry et al, 1991; Schwartz et al, 1991; Walker et al, 1995; Tutschek et al, 1996). The more recent development of reverse transcription polymerase chain reaction (RT-PCR), which may be a more sensitive method to detect pS2, allows both the detection and quantification of pS2 mRNA expression (Carr et al, 1995). It is unclear whether improved sensitivity would effect the prognostic value of pS2 mRNA.

pS2 expression was significantly related to oestrogen receptor protein expression as expected for an oestrogen-regulated protein (Rio et al, 1987; Stack et al, 1988; Skilton et al, 1989; Henry et al, 1991; Schwartz et al, 1991; Predine et al, 1992; Thompson et al, 1993; Detre et al, 1994; Foekens et al, 1994; Speiser et al, 1994; Stonelake et al, 1994; Walker et al, 1995; Gibert et al, 1996; Tutschek et al, 1996) and we have confirmed the observations of others that pS2 expression (whether measured as mRNA or protein) is not significantly associated with tumour size or node status (Foekens et al, 1990; Schwartz et al, 1991; Delvenne et al, 1992; Gion et al, 1993).

We have demonstrated that, at medium-term (6 year minimum, mean 83 months) follow-up, pS2 expression remains associated with good prognosis (Foekens et al, 1990; Schwartz et al, 1991; Predine et al, 1992; Thompson et al, 1993; Gion et al, 1993; Foekens et al, 1994; Speiser et al, 1994; Schmidt et al, 1996) in contrast to one other study of pS2 mRNA with a shorter follow-up (Wysocki et al, 1994). Although our study, like that of Speiser et al (1994), failed to identify pS2 as an independent prognostic factor for survival (which had been suggested by Gion et al, 1993; Foekens et al, 1994), oestrogen receptor protein was associated with survival after adjusting for node status and tumour size. As expected, axillary node status (based on the pathological examination of surgically resected axillary lymph nodes) and tumour size were significantly associated with disease recurrence and survival both on univariate analysis and multivariate analysis.

The most important clinical implication of this study is the confirmation that pS2 expression has a discriminant effect for better prognosis in both axillary lymph node-positive (Foekens et al, 1990; Kausitz et al, 1994) and node-negative patients (Stonelake et al, 1994). Furthermore, the data presented here suggest that a combination of axillary node status and pS2 expression measured at the time of diagnosis can define patients at high and low risk of death for medium-term follow-up.

As pS2 expression is a good indicator of endocrine responsiveness both in the primary cancer and on relapse (Henry et al, 1988; Ramm et al, 1988; Skilton et al, 1989; Schwartz et al, 1991; Westley and May, 1991; Coradini et al, 1996; Soubeyran et al, 1996), the detection of pS2 expression in the primary cancer, whether at the mRNA or the protein level, may be useful both as prognostic information and as a guide to therapeutic intervention. In this study, patients with tumours that were oestrogen receptor moderate/rich at diagnosis (therefore including the tumours that express pS2) were treated with tamoxifen. Thus, as changes in pS2 protein expression have been demonstrated during tamoxifen therapy (Wilsher et al, 1996), it is possible that the apparent survival advantage at 5 years for the patients with pS2 mRNA detected in the tumour may be attributable to the effect of tamoxifen therapy mediated via pS2.

The usefulness of pS2 has been taken one step further in clinical practice; pS2 expression can be used to guide the likely response to tamoxifen therapy in patients with recurrent breast cancer (Foekens et al, 1994). While pS2 expression by Northern blotting may not be suitable for routine pS2 analysis, pS2 measured by immunohistochemical staining (Wilson et al, 1994) or competitive reverse transcription polymerase chain reaction (Carr et al, 1995) on fine-needle aspirates of breast cancer has been used to select patients over the age of 70 years for tamoxifen therapy. pS2 expression in breast cancer tissue is useful in itself as a marker of probable hormonal response to endocrine manipulation. This study has demonstrated that pS2 mRNA expression also provides useful prognostic information in addition to that provided by axillary node status when oestrogen receptor expression may be of limited value. pS2 should now be considered for routine clinical measurement as a response indicator and prognostic marker alongside oestrogen receptor protein.

### ACKNOWLEDGEMENTS

The authors thank Dr Wilma Jack and Mrs Ruby Wood for assistance in collecting follow-up data, the Sarah Percy Fund and Scottish Hospitals Endowments Research Trust (Grant SHERT 868) for financial support and Professor P Chambon for permission to use the pS2 probe.

### REFERENCES

Anderson EDC, Forrest APM, Levack PA, Chetty U and Hawkins RA (1989) Response to endocrine manipulation and oestrogen receptor concentration in large operable primary breast cancer. *Br J Cancer* **60**: 223–226

Carr M, May FEB, Lennard TWJ and Westley BR (1995) Determination of oestrogen responsiveness of breast cancer by competitive reverse transcription polymerase chain reaction. *Br J Cancer* **72**: 1427–1434

Coradini D, Biganzoli E, Boracchi P, Martelli G and Di Fronzo G (1996) Predictive role of steroid receptors pS2 and Cathepsin D on the outcome of elderly breast cancer patients. *Br Can Res Treat* **41**: 249

Delvenne CG, Winkler-Gol RA, Piccart MJ, Hustin J, Michaux D, Leclerecq G, Nogaret JM and Autier P (1992) Expression of c-erbB2, TGF-beta 1 and pS2 genes in primary human breast cancers. *Eur J Cancer* **28**: 700–705

Detre S, King N, Salter S, MacLennan K, McKinna JA and Dowsett M (1994) Immunohistochemical and biochemical analysis of the oestrogen regulated

© Cancer Research Campaign 1998

protein pS2, and its relation with oestrogen receptor and progesterone receptor in breast cancer. *J Clin Pathol* **47**: 240–244

Foekens JA, Rio M-C, Seguin P, Putten WLJ, Van Fauque I, Nap M, Kiljn JGM and Chambon P (1990) Prediction of relapse and survival in breast cancer patients by pS2 protein status. *Cancer Res* **50**: 3832–3837

Foekens JA, Portengen H, Look MP, van Putten WLJ, Thirion B, Bontenbal M and Kiljn JGM (1994) Relationship of pS2 with response to tamoxifen therapy in patients with recurrent breast cancer. *Br J Cancer* **70**: 1217–1223

Gibert OD, Machuca I, Sebastian MA, Rosel P and Navarro MA (1996) pS2 is a useful predictor of regression in tamoxifen therapy. *Med Clin* **107**: 90–92

Gion M, Mione R, Pappagallo GL, Gatti C, Nascimben O, Bari M, Leon AE, Vinante O and Bruscagnin G (1993) pS2 in breast cancer – alternative or complementary tool to steroid receptor status? Evaluation of 446 cases. *Br J Cancer* **68**: 374–379

Hahnel E, Joyce R, Sterrett C, Harvey I and Hahnel R (1991) Detection of estradiol-induced messenger RNA (pS2) in uninvolved breast tissue from mastectomies for breast cancer. *Br Cancer Res Treat* **20**: 167–176

Henry JA, Nicholson S, Farndon JR, Westley BR and May FEB (1988) Measurement of oestrogen receptor mRNA levels in human breast tumours. *Br J Cancer* **58**: 600–605

Henry JA, Nicholson S, Hennessy C, Lennard TWJ, May FEB and Westley BR (1990) Expression of the oestrogen regulated pNR-2 mRNA in human breast cancer: relation to oestrogen receptor mRNA levels and response to tamoxifen therapy. *Br J Cancer* **61**: 32–38

Henry JA; Piggott NH, Mallick UK, Nicholson S, Farndon JR, Westley BR and May FEB (1991) pNR-2/pS2 immunohistochemical staining in breast cancer: correlation with prognostic factors and endocrine response. *Br J Cancer* **63**: 615–622

Kardas I, Seitz G, Limon J, Niezabitowski A, Rys J, Theisinger B, Welter C and Blin N (1993) Retrospective analysis of prognostic significance of the estrogen-inducible pS2 gene in male breast cancer. *Cancer* **72**: 1652–1656

Kausitz J, Kuliffay P, Pecen L, Eben K and Puterova B (1994) Correlation of cytosolic concentrations of ER, PS2, Cath-D, TPS, TK and cAMP in primary breast carcinomas. *Neoplasma* **41**: 331–336

Masiakowski P, Breathnach R, Bloch I, Gannon F, Krust A and Chambon P (1982) Cloning of cDNA sequences of hormone-regulated genes from the MCF-7 human breast cancer cell line. *Nucleic Acids Res* **10**: 7895–7903

Minty A, Caravatti M, Robert B, Cohen A, Daubas P, Weydert A, Gros F and Buckingham ME (1981) Mouse actin messenger RNAs. *J Biol Chem* **256**: 1008–1014

Predine I, Spyratos F, Prud-Homme JF, Andrieu C, Hacene K, Brunet M, Pallud C and Milgrom E (1992) Enzyme-linked immunosorbent assay of pS2 in breast cancers, benign tumours, and normal breast tissues. *Cancer* **69**: 2116–2123

Ramm S, Robert N, Pappas CA and Tamura H (1988) Defective estrogen receptors in human mammary cancers: their significance in defining hormone dependence. *J Natl Cancer Inst* **80**: 756–761

Rio MC, Bellocq JP, Gairard B, Rasmussen UB, Krust A, Koehl C, Calderoli H, Schiff V, Renaud R and Chambon P (1987) Specific expression of the pS2 gene in subclasses of breast cancers in comparison with expression of the estrogen and progesterone receptors and the oncogene ERBB2. *Proc Natl Acad Sci USA* **84**: 9243–9247

Schmidt R, Sorger D, Walter F, Schonfelder M and Preiss R (1996) pS2 protein, EGFR and Cathepsin-D in association with established prognostic factors in early recurrence of breast cancer. *Onkologie* **19**: 176–180

Schwartz LH, Koerner FC, Edgerton SM, Sawicka JM, Rio MC, Bellocq JP, Chambon P and Thor AD (1991) pS2 expression and response to hormonal therapy in patients with advanced breast cancer. *Cancer Res* **51**: 624–628

Skilton RA, Luqmani YA, McClelland RA and Coombes RC (1989) Characterisation of a messenger RNA selectively expressed in human breast cancer. *Br J Cancer* **60**: 168–175

Soubeyran I, Quenel N, Mauric L, Durand M, Bonichon F and Coindre J-M (1996) Variation of hormonal receptor, pS2, c-erbB-2 and GSTpi contents in breast carcinomas under tamoxifen: a study of 74 cases. *Br J Cancer* **73**: 735–743

Speiser P, Stoizlechner S, Haider K, Heinzi H, Jakesz R, Pecherstorfer M, Rosen H, Sevelda P and Zeilliger R (1994) pS2 protein status fails to be an independent prognostic factor in an average breast cancer population. *Anticancer Res* **14**: 2125–2130

Stack G, Kumar V, Green S, Ponglikitmongkol M, Berry M, Rio MC, Nunez AM, Roberts M, Bellocq P, Gairard B, Renaud R and Chambon P (1988) Structure and function of the pS2 gene and estrogen receptor in human breast cancer cells. In *Breast Cancer: Cellular and Molecular Biology*, Lippmann ME and Dickson RB. (eds), pp. 185–206. Kluwer: Boston

Stonelake PS, Baker PG, Gillespie WM, Dunn JA, Spooner D, Morrison JM, Bundred NJ, Oates GD, Lee MJ and Neoptolemos JP (1994) Steroid receptors, p52 and cathepsin D in early clinically node-negative breast cancer. *Eur J Cancer* **30**: 5–11

Thompson AM, Hawkins RA, Elton RA, Steel CM, Chetty U and Carter DC (1993) pS2 is an independent factor of good prognosis in primary breast cancer. *Br J Cancer* **68**: 93–96

Tutschek B, Niederacher D, Caminada MC, Gohring UJ, Schaol A, Schnurch HG, Beckman MW and Bender HG (1996) pS2 protein detected by immunohistochemistry in archival primary breast cancer – prognostic value and long term follow-up in 219 patients. *Oncol Rep* **3**: 759–764

Walker RA, Rajakariar R and Dookeran KA (1995) Comparison of pS2 and cathepsin D in primary breast carcinomas. *The Breast* **4**: 137–142

Westley B and May FEB (1991) Estrogen-related messenger RNAs in human breast cancer cells. *Cancer Treat Res* **53**: 259–271

Willsher PW, Gee JMW, Blamey RW, Nicolson RI and Robertson JFR (1996) Changes in ER, PgR and pS2 protein during tamoxifen therapy for primary breast cancer. *Br Cancer Res Treat* **41**: 288

Wilson YG, Rhodes M, Ibrahim NBN, Padfield CJH and Cawthorn SJ (1994) Immunocytochemical staining of ps2 protein in fine-needle aspirate from breast cancer is an accurate guide to response to tamoxifen in patients aged over 70 years. *Br J Surg* **81**: 1155–1158

Wysocki SJ, Iacopetta BJ and Ingram DM (1994) Prognostic significance of pS2 mRNA in breast cancer. *Eur J Cancer* **30A**: 1882–1884

Zaretsky JZ, Weiss M, Tsarfaty I, Hareuveni M, Wreschner DH and Keydar I (1990) Expression of genes coding for pS2, c-erbB2, estrogen receptor and the H23 breast tumour associated antigen. *FEBS Lett* **265**: 46–50

© Cancer Research Campaign 1998


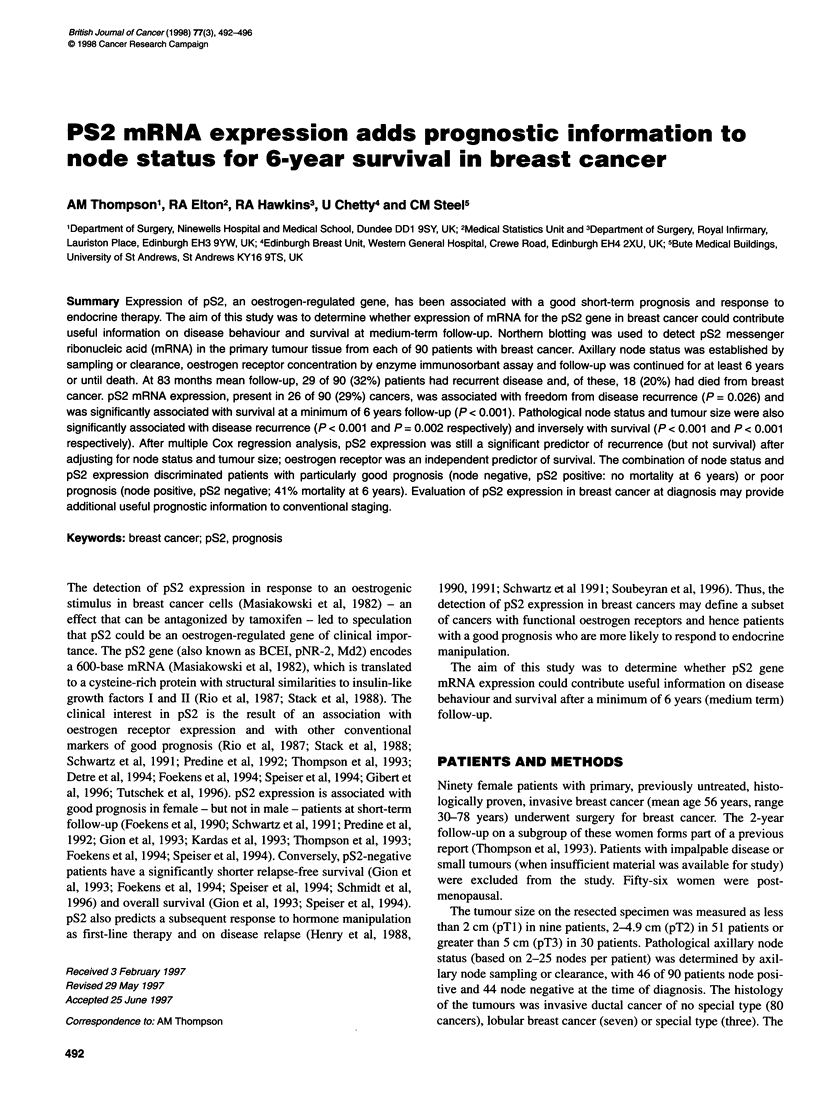

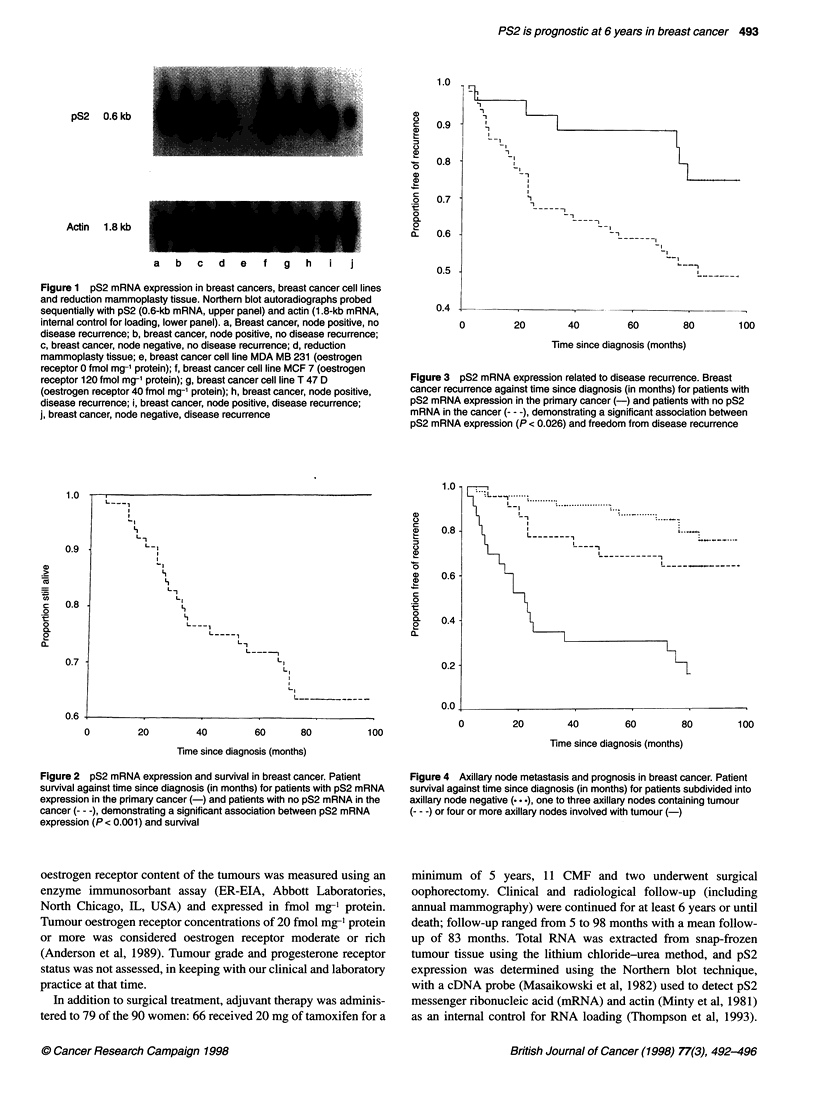

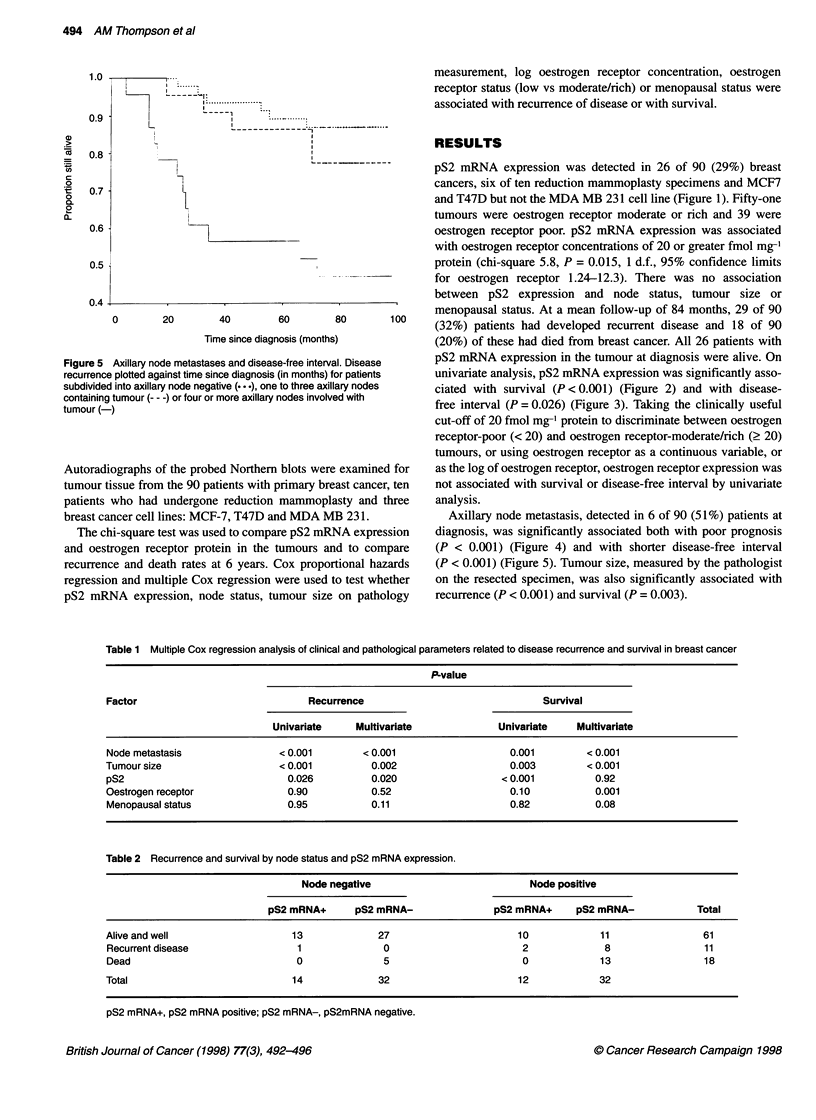

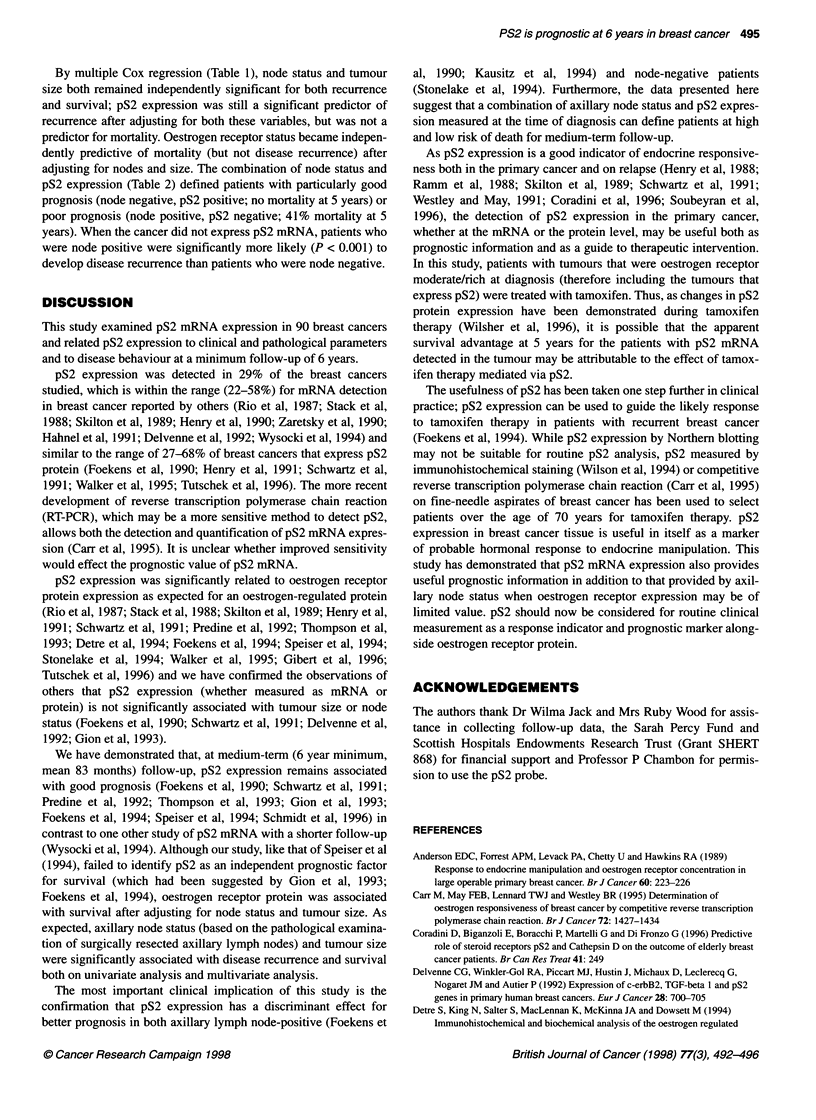

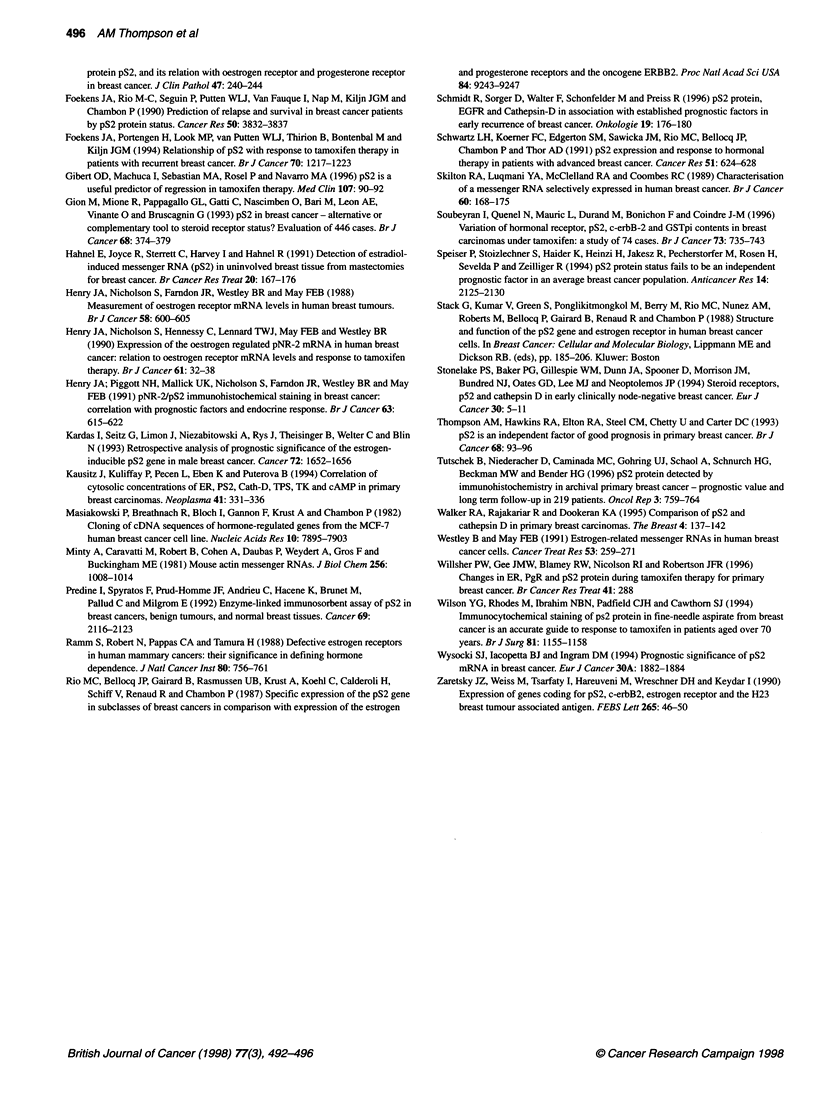


## References

[OCR_00528] Anderson E. D., Forrest A. P., Levack P. A., Chetty U., Hawkins R. A. (1989). Response to endocrine manipulation and oestrogen receptor concentration in large operable primary breast cancer.. Br J Cancer.

[OCR_00533] Carr M., May F. E., Lennard T. W., Westley B. R. (1995). Determination of oestrogen responsiveness of breast cancer by competitive reverse transcription-polymerase chain reaction.. Br J Cancer.

[OCR_00543] Delvenne C. G., Winkler-Gol R. A., Piccart M. J., Hustin J., Michaux D., Leclercq G., Nogaret J. M., Autier P. (1992). Expression of c-erbB2, TGF-beta 1 and pS2 genes in primary human breast cancers.. Eur J Cancer.

[OCR_00548] Detre S., King N., Salter J., MacLennan K., McKinna J. A., Dowsett M. (1994). Immunohistochemical and biochemical analysis of the oestrogen regulated protein pS2, and its relation with oestrogen receptor and progesterone receptor in breast cancer.. J Clin Pathol.

[OCR_00569] Díez Gibert O., Machuca I., Sebastián M. A., Rosel P., Navarro M. A. (1996). Expresión de la proteína pS2 en el cáncer de mama y su relación con los receptores de estrógenos y progesterona.. Med Clin (Barc).

[OCR_00564] Foekens J. A., Portengen H., Look M. P., van Putten W. L., Thirion B., Bontenbal M., Klijn J. G. (1994). Relationship of PS2 with response to tamoxifen therapy in patients with recurrent breast cancer.. Br J Cancer.

[OCR_00559] Foekens J. A., Rio M. C., Seguin P., van Putten W. L., Fauque J., Nap M., Klijn J. G., Chambon P. (1990). Prediction of relapse and survival in breast cancer patients by pS2 protein status.. Cancer Res.

[OCR_00572] Gion M., Mione R., Pappagallo G. L., Gatti C., Nascimben O., Bari M., Leon A. E., Vinante O., Bruscagnin G. (1993). PS2 in breast cancer--alternative or complementary tool to steroid receptor status? Evaluation of 446 cases.. Br J Cancer.

[OCR_00584] Henry J. A., Nicholson S., Farndon J. R., Westley B. R., May F. E. (1988). Measurement of oestrogen receptor mRNA levels in human breast tumours.. Br J Cancer.

[OCR_00589] Henry J. A., Nicholson S., Hennessy C., Lennard T. W., May F. E., Westley B. R. (1990). Expression of the oestrogen regulated pNR-2 mRNA in human breast cancer: relation to oestrogen receptor mRNA levels and response to tamoxifen therapy.. Br J Cancer.

[OCR_00596] Henry J. A., Piggott N. H., Mallick U. K., Nicholson S., Farndon J. R., Westley B. R., May F. E. (1991). pNR-2/pS2 immunohistochemical staining in breast cancer: correlation with prognostic factors and endocrine response.. Br J Cancer.

[OCR_00579] Hähnel E., Joyce R., Sterrett G., Harvey J., Hähnel R. (1992). Detection of estradiol-induced messenger RNA (pS2) in uninvolved breast tissue from mastectomies for breast cancer.. Breast Cancer Res Treat.

[OCR_00603] Kardaś I., Seitz G., Limon J., Niezabitowski A., Ryś J., Theisinger B., Welter C., Blin N. (1993). Retrospective analysis of prognostic significance of the estrogen-inducible pS2 gene in male breast carcinoma.. Cancer.

[OCR_00608] Kausitz J., Kuliffay P., Pecen L., Eben K., Puterová B. (1994). Correlation of cytosolic concentrations of ER, PS2, Cath-D, TPS, TK and cAMP in primary breast carcinomas.. Neoplasma.

[OCR_00613] Masiakowski P., Breathnach R., Bloch J., Gannon F., Krust A., Chambon P. (1982). Cloning of cDNA sequences of hormone-regulated genes from the MCF-7 human breast cancer cell line.. Nucleic Acids Res.

[OCR_00618] Minty A. J., Caravatti M., Robert B., Cohen A., Daubas P., Weydert A., Gros F., Buckingham M. E. (1981). Mouse actin messenger RNAs. Construction and characterization of a recombinant plasmid molecule containing a complementary DNA transcript of mouse alpha-actin mRNA.. J Biol Chem.

[OCR_00623] Predine J., Spyratos F., Prud'homme J. F., Andrieu C., Hacene K., Brunet M., Pallud C., Milgrom E. (1992). Enzyme-linked immunosorbent assay of pS2 in breast cancers, benign tumors, and normal breast tissues. Correlation with prognosis and adjuvant hormone therapy.. Cancer.

[OCR_00629] Raam S., Robert N., Pappas C. A., Tamura H. (1988). Defective estrogen receptors in human mammary cancers: their significance in defining hormone dependence.. J Natl Cancer Inst.

[OCR_00634] Rio M. C., Bellocq J. P., Gairard B., Rasmussen U. B., Krust A., Koehl C., Calderoli H., Schiff V., Renaud R., Chambon P. (1987). Specific expression of the pS2 gene in subclasses of breast cancers in comparison with expression of the estrogen and progesterone receptors and the oncogene ERBB2.. Proc Natl Acad Sci U S A.

[OCR_00647] Schwartz L. H., Koerner F. C., Edgerton S. M., Sawicka J. M., Rio M. C., Bellocq J. P., Chambon P., Thor A. D. (1991). pS2 expression and response to hormonal therapy in patients with advanced breast cancer.. Cancer Res.

[OCR_00652] Skilton R. A., Luqmani Y. A., McClelland R. A., Coombes R. C. (1989). Characterisation of a messenger RNA selectively expressed in human breast cancer.. Br J Cancer.

[OCR_00657] Soubeyran I., Quénel N., Mauriac L., Durand M., Bonichon F., Coindre J-M (1996). Variation of hormonal receptor, pS2, c-erbB-2 and GSTpi contents in breast carcinomas under tamoxifen: a study of 74 cases.. Br J Cancer.

[OCR_00662] Speiser P., Stolzlechner J., Haider K., Heinzl H., Jakesz R., Pecherstorfer M., Rosen H., Sevelda P., Zeilliger R. (1994). pS2 protein status fails to be an independent prognostic factor in an average breast cancer population.. Anticancer Res.

[OCR_00676] Stonelake P. S., Baker P. G., Gillespie W. M., Dunn J. A., Spooner D., Morrison J. M., Bundred N. J., Oates G. D., Lee M. J., Neoptolemos J. P. (1994). Steroid receptors, pS2 and cathepsin D in early clinically node-negative breast cancer.. Eur J Cancer.

[OCR_00682] Thompson A. M., Hawkins R. A., Elton R. A., Steel C. M., Chetty U., Carter D. C. (1993). pS2 is an independent factor of good prognosis in primary breast cancer.. Br J Cancer.

[OCR_00698] Westley B., May F. E. (1991). Estrogen-regulated messenger RNAs in human breast cancer cells.. Cancer Treat Res.

[OCR_00707] Wilson Y. G., Rhodes M., Ibrahim N. B., Padfield C. J., Cawthorn S. J. (1994). Immunocytochemical staining of pS2 protein in fine-needle aspirate from breast cancer is an accurate guide to response to tamoxifen in patients aged over 70 years.. Br J Surg.

[OCR_00713] Wysocki S. J., Iacopetta B. J., Ingram D. M. (1994). Prognostic significance of pS2 mRNA in breast cancer.. Eur J Cancer.

[OCR_00717] Zaretsky J. Z., Weiss M., Tsarfaty I., Hareuveni M., Wreschner D. H., Keydar I. (1990). Expression of genes coding for pS2, c-erbB2, estrogen receptor and the H23 breast tumor-associated antigen. A comparative analysis in breast cancer.. FEBS Lett.

